# Exploring the positive aspects of caregiving among family caregivers of the older adults in India

**DOI:** 10.3389/fpubh.2023.1059459

**Published:** 2023-05-18

**Authors:** Nidhi Mishra, Ravi S. Datti, Ashutosh Tewari, Maneela Sirisety

**Affiliations:** ^1^Department of Applied Psychology, School of Humanities and Social Sciences, Gandhi Institute of Technology and Management (GITAM), Visakhapatnam, India; ^2^Venture Development Center, Gandhi Institute of Technology and Management (GITAM), Visakhapatnam, India

**Keywords:** aging, positive caregiving, informal caregivers, family caregivers, well-being, older adults, India, qualitative methods

## Abstract

**Background:**

Caregiving is a committed role that focuses on providing the required support and care to a care receiver who is either ill, disabled, or dependent to the extent that they are incapable of independent functioning. The topic of caregiving has been extensively studied worldwide, mainly focused on the negative aspects of caregiving, like caregiver stress, burden, role conflicts, and burnout among caregivers. However, limited efforts have been made to understand the positive aspects of caregiving among informal caregivers who spend most of their time in this unpaid role. The present study addresses this concern by exploring the positive aspects of caregiving among family caregivers of older persons in India.

**Methodology:**

This is a qualitative study, which was conducted, using the existential-phenomenological approach. In this study, a purposive sampling technique was used, and a total of a 100 family caregivers participated from four cities in India, namely Prayagraj, Pune, Visakhapatnam, and Guwahati. Twenty-five family caregivers between the age of 30–50  years participated from each of these four cities. The data was collected using six semi-structured interview questions on positive aspects of caregiving based on the lived experiences of caregivers. The interview schedule was developed based on the PERMA model and finalized after the pilot study. Each interview took 30–50 min and was recorded and transcribed.

**Results:**

The data was analyzed using thematic analysis. Some commonalities and differences were observed in the responses given by family caregivers from the four cities. Four major themes that emerged for the “Nature of positive caregiving” among participants from all four cities are “Caregiver’s attitude,” “Care and compassion,” “Roles and responsibilities,” and “Beliefs and values.” Four significant themes for the “Factors contributing to positive caregiving” are “Meaningfulness in life,” “Sense of belongingness,” “Personal growth,” and “Empathetic understanding.”

**Conclusion:**

Based on the study’s findings, it can be said that the themes were associated with the PERMA model. Positive caregiving is an important indicator of caregivers’ and care receivers’ well-being. The findings have implications for planning some action research, training, and counseling programs for promoting positive aspects of caregiving among informal caregivers.

## 1. Introduction

The older adults population is increasing at a fast pace throughout the world. India is the second most older adults populated country globally, with 103.8 million senior citizens (1, 2). The need for formal and informal caregivers is subject to increase here at the same pace to maintain the holistic health of older people. Being a collectivistic country, it is an age-old tradition in India that family members look after the older adults. Though the changes in certain demographic and care-specific factors are making family members opt for formal caregiving, the preference for informal caregiving, especially the caregiver being the spouse or child, is still high compared to formal ones (3–5).

Informal caregiving is a dynamic, evolving, and intersubjective life experience in which connections are co-created consciously or unconsciously and manifested in acts and behaviors of care, responsibility, and attention (6). Caregiving is often associated with many positive and negative aspects depending upon every stakeholder and the internal and external factors involved (3, 7, 8). Given its dynamic nature, researchers must study these factors and their associations with each other and plan necessary interventions that promote the positive well-being of both the care-receivers and caregivers (9).

Looking into the literature, it is observed that many researchers have focused on the negative side of caregiving and have highlighted factors such as burden, stress, clinical depression, cognitive impairment, physical health problems, and burnout commonly observed in formal and informal caregivers (10–14). However, limited attention is given to exploring the positive aspects of caregiving (8, 15–18). Given this gap and the necessity to understand the positive side of caregiving, the current study aims to highlight the positive factors associated with the caregiving of the older adults.

Perceptions of caregiving and the factors influencing caregivers differ across cultures (19). A study by EUROFAMCARE in Europe found different factors influencing informal caregivers’ decision to be involved in caregiving (20). According to this study, emotional bonding with the care-receiver mostly prompted the informal caregivers to provide caregiving. On the positive side, a tiny percentage of caregivers considered caregiving as a sense of obligation and taking up the role because there are no other alternatives.

In the Indian context, caregiving is usually provided by family members. Additionally, extended family, friends, and neighbors feel responsible for caring for older people (3). However, due to many factors, caregiving is perceived in mixed ways. Some find it rewarding to serve their elders and view it positively, whereas others find it burdensome (14). For instance, a study conducted in Visakhapatnam (Andhra Pradesh, India) indicated that though caregiving is satisfactory, poor health conditions and tiredness are reported by women caregivers due to increased stress arising from their caregiving roles (21). Similar results were reported by a study conducted in Pune (Maharashtra, India). This study highlights that providing care to older adults parents gave the caregivers eternal satisfaction and led to deep and rewarding emotional bonds among the family members. However, the study also reported that children try to avoid the responsibility of providing care to their older adults parents as carrying out this act involves constant physical and mental effort, in addition to facing high financial costs (7). Looking into these differences in perceptions, it can be understood that cultural factors play one of the critical roles in deciding the caregiver’s attitude towards caregiving.

Some of the studies also point out that individual factors also determine the caregiver’s attitude, along with cultural factors. A systematic review (22) carried out to look at role of psychosocial (self-esteem, resilience, family relationships, and financial status) and individual factors (good relationship with care-receiver, social support, and personality) in caregiving indicated that caregivers who are aged more than 65 years, experience more burden and physical health issues. However, psychological factors like self-esteem and resilience can attenuate the negativity associated with caregiving and act as protective factors. It is essential to highlight the fact that though most of the caregivers are women who are in their 40s and have reported more distress, strain, and burden than men, studies point out that only a small part of the variation in the caregiving outcomes may be attributed to gender (8, 23).

The literature shows that the researchers’ focus over the last decade has shifted to looking into caregiving outcomes by considering different internal and external factors. Irrespective of the type of care, i.e., providing care to the person with or without physical or psychological or multiple impairments (including activities of daily living, cognition, vision, and hearing), caregivers of older adults experience moderate to higher levels of burden due to factors like characteristics of the care-receivers and their illness, socioeconomic status, caregivers’ physical health, psychopathological symptoms, and disruption of the family routine (9, 12, 14, 24, 25).

Studies have also pointed out that caregivers of patients with psychiatric and chronic medical illnesses risk experiencing burnout, dysphoria, emotional distress, stress, anxiety, and depression, even though the burnout levels are moderate (10, 11, 25–28). These experiences impact the caregivers’ physical and psychological health, social relationships, and environmental interactions, leading to low quality of life. However, these experiences differ among males and females and are subject to change depending on the care-receiver’s age and illness.

From the above discussion, it can be inferred that individual differences have an essential role in the caregiving outcome, and caregiving generally depends on how well the caregivers adjust to the demands of caregiving. The government and policymakers must consider these cultural and individual differences while framing any policies and schemes. The government must support and facilitate the informal caregivers by teaching them problem-focused coping strategies, providing home health visit services, and identifying different types of needs (like organizational, informational, support, and societal recognition) so that these facilities mitigate the possible adverse effects associated with caregiving (4, 14, 29).

Although most studies have highlighted the problematic side of caregiving, some researchers have also investigated the positive outcomes associated with caregiving. When the caregivers have a good relationship with the care-receiver, they choose to provide care out of their own will, allocate some time for self-care, vent out emotions occasionally, and are not working out of the home; they have been shown to exhibit satisfaction in their caregiving (8, 16). Having a ‘feeling of accomplishment,’ being appreciated and respected by the care receiver, taking an active role in daily enrichment events such as going out for a walk, taking rest, and privately grieving away from the patient were found to be some other determining factors in positive caregiving (15, 18). In addition to these factors, resilience and a sense of coherence were demonstrated to be positively related to the satisfaction associated with caregiving when the caregivers develop a sense of commitment to caregiving by assigning a high value to the events in their lives (17). In the Indian context, along with the factors mentioned above, social support, being hopeful, and having religious faith were also shown to promote positive aspects of caregiving (15).

From the above studies, it is evident that a limited number of studies have been done to explore the positive aspects of caregiving. Given this gap, the researchers must explore the flourishing aspects of caregiving and come to a consensus on the domain of factors associated with it so that they can be nurtured and act as preventative factors against any potential negative consequences of caregiving. Given the need, the current study aims to understand the nature of positive caregiving and explore the factors associated with such experiences among informal caregivers using the PERMA model.

PERMA model is a multi-dimensional approach to defining what it means to flourish in life. It was developed by Martin Seligman (30) to define psychological well-being in five domains, Positive Emotion (P), Engagement (E), Relationship (R), Meaning (M), and Accomplishment (A). This framework was utilized in various contexts across cultures and has proven to be a good measure of well-being (31, 32).

According to the PERMA model, Positive emotions (P) comprise hope, interest, joy, love, compassion, pride, amusement, and gratitude. They are essential indicators of flourishing and well-being (33). Studies have highlighted that including positive emotions in daily life can lead to positive thinking, reduce negative emotions, and promote resilience (34). This model’s second domain, Engagement (E), emphasizes living in the present moment and focusing entirely on the task at hand. Studies on engagement reported that individuals using strengths in new ways each day while engaging in tasks for a week were happier and less depressed after 6 months (35). Relationships (R) in the PERMA model refers to feeling supported, loved, and valued by others. Studies have indicated that sharing good news or celebrating success fosters solid bonds and better relationships (36).

In the PERMA model, Meaning (M) is associated with having a purpose in life and is guided by personal values. Studies have highlighted that having a purpose in participants’ lived experiences reduces health problems (37). It has also been found that getting involved in a cause that matters can bring more meaning in life (38). Accomplishment (A) in PERMA is also known as achievement, mastery, or competence. It has been found that flourishing and well-being will be experienced when accomplishment is linked to attaining intrinsic goals such as growth and connection (30).

To date, limited studies have been conducted by researchers who looked at the caregiving of the older adults using the PERMA model. Also, the essence of flourishing in caregivers can be understood well qualitatively based on their lived experiences; however, only a few studies have focused on it in this way. The current study incorporated the PERMA Model as it is an evidence-based approach giving a scientific base to exploring flourishing among caregivers based on their positive caregiving lived experiences. Using the PERMA model, the present study aims to understand the nature of positive caregiving and explore the factors contributing to positive caregiving experiences among family caregivers of older adults in India based on the existential-phenomenological approach.

## 2. Research design and methods

This study is a qualitative study that was conducted using the existential-phenomenological approach. The approach views people as being in a constant process of interpretation as they journey from one situation to another. Each situation has meaning only through people’s interpretation and definition of it. According to this approach, one must understand a person’s experience from their lifeworld. It is the world as lived by the person every day in direct and immediate experience (39). In the present study, efforts were made to understand the lifeworld of the caregivers through their everyday caregiving experiences in the context of their caregiving role.

### 2.1. Participants

In this study, a purposive sampling technique was used, and a total of 100 family caregivers from four cities in India, namely Prayagraj, Pune, Visakhapatnam, and Guwahati participated in this study.

These cities were selected as they have a sizeable population of older adults and because they have an increased number of informal caregivers due to the limited availability of paid caregiving services. Prayagraj from Uttar Pradesh represented North India, Visakhapatnam from Andhra Pradesh represented South India, Pune from Maharashtra is in Western India, and Guwahati is in the Eastern part of India. Twenty-five middle-income family caregivers aged 30–50 years, participated from each of these four cities. From Prayagraj, 19 female caregivers and 6 male caregivers; from Visakhapatnam, 21 female caregivers, and 4 male caregivers; from Pune, 16 female caregivers, and 9 male caregivers; and from Guwahati, 18 female caregivers and 7 male caregivers participated in this study. Across the four cities, the female caregivers were mostly daughters-in-law and daughters, and the male caregivers were sons taking care of their older adults parents.

### 2.2. Data collection

The data was collected using the interview technique. Six semi-structured interview questions on positive aspects of caregiving based on participants’ lived experiences were asked from each participant. The interview schedule was developed based on the PERMA model. The six questions were focused on exploring positive emotions, engagement, relationships, meaning, and accomplishment of caregivers in their caregiving roles. They are as follows:

- Discuss how your role of caregiving has impacted your life.
- Share some of your positive emotional experiences associated with your role as a caregiver.
- How engaged and involved do you find yourself in your caregiver’s role?
- What type of relationship do you share with your care receiver?
- How and in what ways has your role as a caregiver defined the meaning of your life?
- What type of satisfaction did you get in this role of caregiver? Share some of your accomplishments.

The interview schedule was tested and finalized after the pilot study, which was done in all four cities.

The data collection was done in four phases. Each phase took a month. In phase one, data was collected in Prayagraj; in phase two, data was collected in Visakhapatnam; in phase three, data was collected in Pune; in phase four, data was collected in Guwahati. Each interview in the final study took 30–50 min and was recorded and transcribed. Interviews were primarily conducted in Hindi and English in all four cities. In Visakhapatnam, some interviews were conducted in Telugu, and in Guwahati, some were conducted in Assamese.

To maintain this study’s reliability, before data collection, the researchers practiced bracketing by first writing their preconceptions about family caregiving. This was done to go for data collection with an open mind for accepting participants’ experiences without any preconceived notions. In practice, bracketing is not so easy and difficult to follow; however, the researchers tried to become aware of their own ideas regarding informal caregiving and not to let those ideas interfere in data collection and analysis.

To establish the validity in the present research study, the following strategies were applied:

Prolonged engagement in the field: the researcher’s prolonged engagement in the field is an important strategy for establishing validity. According to experts (40), during repeated observations, the researcher builds trust with the participants, establishes rapport so that participants feel comfortable, and thus discloses information. Covering a small population over a long period enables a holistic and in-depth understanding of reality (41). In the present research, researchers applied this strategy by visiting the field more than once in each of the four cities to establish rapport with participants and to collect data. During these visits, researchers stayed there for a, more extended period, not only for data collection but to establish rapport with participants by having a general conversation to make them feel at ease before the data collection.

Theoretical sampling: theoretical sampling refers to the process of selecting informants who represent the community, not statistically, but theoretically, in the sense of having more knowledge about the community for which the study is designed or any specific aspect of the community life (42). This strategy has been applied in this study by collecting data from informal caregivers residing and practicing their caregiving role in the four cities.

Extensive quotations: extensive quotations from field notes and transcripts of the interviews are also an essential way of establishing credibility in qualitative research (43). This strategy has been used in this study by quoting the participants’ responses from the transcripts while discussing the study results (for details, please see the result and discussion section).

Member checking: member checking has been described as the most crucial technique for establishing credibility (44). It consists of taking data and interpretations back to the participants so that they can confirm the credibility of the information and narrative accounts. This strategy has been applied in this research by taking transcriptions back to the respective participants in the case of all four cities and asking them to check or add any further information to it. Some of the responses which were not very clear and which needed further clarity were also checked.

Multiple researchers checking the data: it involves more than one researcher reviewing the data and its interpretation (43). This is done for verification of data and its interpretations by more than one researcher. In the present research, this strategy has been followed. Data, themes, and interpretations were checked and verified by all the authors.

After the checking of transcripts by researchers and participants the transcribed responses were treated as final data for the analysis.

### 2.3. Data analysis

The data was analyzed using thematic analysis.

The thematic analysis of the responses of participants from each of the four cities was done separately but simultaneously. In the case of each city’s data, thematic analysis was done flexibly following six steps stated by Braun and Clarke (45). These steps are discussed in detail below:

Step 1: familiarizing oneself with the data—this was the first step of data analysis. In this step, the researchers transcribed all 25 interviews collected in the case of each city. After that, all 25 transcriptions were read and re-read by the researchers one by one, specifically for each of the six semi-structured questions asked in case of each interview. Based on the readings of each transcription, some ideas related to each question were identified.
Step 2: generating initial codes—in this step, based on the ideas noted in the earlier step, codes were generated. Codes identify a feature of the data that appears significant to the analyst and refer to the distinctive features of the raw data that can be analyzed in a meaningful way regarding the phenomenon (46). The coding process is part of the analysis (42), as one organizes data into meaningful groups (47). In the present research study, coding is theory-driven, and the thematic analysis was done based on the existential-phenomenological approach and the PERMA model.
Separate coding for each of the six questions in each participant’s case from four cities was done. Though coding was done based on the questions asked, if any participant gave any response which was not directly related to the question but the participant confirmed it to be the answer for the same question, then it was also noted because being a phenomenological study any response from the participant was considered significant.
Step 3: searching for themes—in this step, two separate lists of codes, one for the nature of positive caregiving and another one for factors related to positive caregiving, were prepared, which were extracted under the respective six questions in the earlier step from each of the 25 interviews. After this, based on those codes, themes were identified for nature and factors related to positive caregiving.
Step 4: reviewing themes—in this step, the themes were refined, which were prepared in the earlier step. During this review phase, the researchers combined some sub-themes and made them into one theme.
Step 5: defining and naming themes—in this step based on the ‘essence’ of what each theme is about and what aspect of the data each theme captures, all the themes generated were defined and named.
Step 6: producing the report—after the final themes were named and defined, a write-up containing the description of each theme (with responses) was prepared, which is a part of this study’s result and discussion chapter.

Though these steps are mentioned separately, in practice, the researchers often worked simultaneously on reading transcripts, preparing codes, making themes based on codes, and reviewing themes. The same procedure was followed for the data in all four cities.

## 3. Results

Based on the thematic analysis of the responses given by the 100 family caregivers in this study, four major themes that emerged for the “Nature of positive caregiving” are “Caregiver’s attitude,” “Care and compassion,” “Roles and responsibilities,” and “Beliefs and values.”

### 3.1. Nature of positive caregiving

#### 3.1.1. Caregiver’s attitude

Caregiver’s attitude was the dominant theme among participants from all four cities. The respondents emphasized that since caregiving has brought meaningfulness to their life, they want to give their best in this role by serving the care receivers with the utmost respect. Most of the participants from Pune and Guwahati mentioned that taking care of older adults family members is like expressing one’s gratitude for what they did for these caregivers when they were young. Like a caregiver (Daughter, 35 years) from Pune said, “This caregiving role gives me lots of satisfaction as I am serving my father who fulfilled all my wishes when I was a kid.” Another caregiver (Son, 40 years) from Guwahati mentioned, “When I was a child, my mother took good care of me, and now it is my responsibility to give back through my caregiving role.” Most caregivers from Prayagraj and Visakhapatnam emphasized fulfilling their caregiving role with utmost seriousness to inculcate positive values in their younger generation. A caregiver from Prayagraj (Daughter-in-law, 39 years) said, “I am doing my caregiving duty for my mother-in-law with all my heart so that my children should learn such good values now and perform such roles when required.” Similarly, a caregiver (Daughter-in-law, 35 years) from Visakhapatnam said, “I am glad that I am able to fulfill my caregiving role with dedication which I am sure will inspire my children to learn good values like taking care of older adults parents and grandparents.”

#### 3.1.2. Care and compassion

Care and compassion was the second dominant theme among respondents from all four cities. The respondents emphasized that caregiving is a pious job as the caregiver not only takes care of the care receiver but also motivates them when they feel low due to illness and disability. In this way, they raise the care receiver’s self-worth through their caring behavior and compassionate service. Like a caregiver from Prayagraj (Daughter, 40 years) said, “Whenever my mother feels low because of her illness, I try to cheer her up. It makes me feel good.” Another caregiver from Visakhapatnam (Son, 50 years) said, “My father is 80, and sometimes he feels low because of his mobility issues; during that time as a son and caregiver, I encourage him to think positively.” Similarly, a caregiver from Guwahati (Daughter-in-law, 38 years) said, “I think caregiver’s role is a very holy job as in this way we help someone in need.” Some caregivers from Pune further shared their experiences of raising the care receiver’s confidence level through their care and determination. Like a caregiver (Son, 42 years) mentioned, “Whenever my parents want to go for long walks, it is hard for them to do that; in that situation, I join them and boost their self-confidence.”

#### 3.1.3. Roles and responsibilities

Most participants across the four cities emphasized that the caregiving role and responsibility gives a purpose to their life through engagement and involvement. According to caregivers, this brings satisfaction in their life and makes the family bond stronger. Like a respondent from Pune (Daughter-in-law, 37 years) said, “Serving my parents-in-law is my main responsibility; this work gives me satisfaction and keeps me busy.” Another caregiver from Prayagraj (Daughter-in-law, 35 years) said, “This caregiving responsibility adds more importance to my life. I will fulfill my responsibility with utmost sincerity.” Some respondents from Guwahati and Visakhapatnam emphasized that this role has strengthened their family bonding. Like a caregiver from Guwahati (Daughter, 44 years) said, “I am satisfied that I am in a position to take care of my mother; this is strengthening our bonding.” Another one from Visakhapatnam (Son, 45 years) said, “I am glad that I am taking care of my parents in their old age, it is making our relationship stronger.”

#### 3.1.4. Beliefs and values

This theme revolves around caregivers’ belief that their caregiving role contributes to their spiritual development and family welfare. It also highlights the importance or value they give to their caregiving role, which is bringing good deeds (Karma). Here most participants from Prayagraj and Pune mentioned that their caregiving role is helping them to attain their spiritual development. Like a caregiver from Prayagraj (Daughter-in-law, 46 years) said, “Taking care of elders is a holy job which is helping me in my spiritual growth.” Another caregiver from Pune (Daughter-in-law, 49 years) said, “This caregiving is a pious work and a means to spiritual attainment.” Some participants from Guwahati believed it led to the building of intergenerational bonding in the family. Like a participant (Daughter, 41 years) said, “Due to this role, I have become closer to my parents, and my children have become closer to me. We all respect and love each other.” Some others in Visakhapatnam related it to good karma. Like a participant (Daughter-in-law, 39 years) said, “I am sure these deeds of mine in the form of caregiving will bring good karma for my family and me.”

### 3.2. Factors contributing to positive caregiving

Based on the thematic analysis, the four significant themes that emerged as factors contributing to positive caregiving are “Meaningfulness in life,” “Sense of belongingness,” “Personal growth,” and “Empathetic understanding.” Here also, some commonalities and differences were observed in the responses given by participants across four cities.

#### 3.2.1. Meaningfulness in life

In the case of the theme “Meaningfulness in life,” participants from Prayagraj, Visakhapatnam, and Guwahati derived meaningfulness in their work and life because of the sense of satisfaction and fulfillment they experienced in this role. Thus, highlighting meaningfulness and purposefulness is an essential factor in caregivers’ positive caregiving experiences. Like a participant from Prayagraj said (Son, 48 years), “This duty of mine towards my parents gives a purpose to my life.” Another caregiver from Visakhapatnam (Daughter, 38 years) said that “I am satisfied that I am taking care of my parents in their old age. It adds meaning to my life.” Similarly a caregiver from Guwahati (Daughter-in-law, 35 years) said, “This caregiving role is tough, but I am glad that my life is useful for someone.” Most participants from Pune equated it more with positive self-worth. Like a caregiver (Daughter-in-law, 40 years) said, “My caregiving role adds more value to my life and makes my life meaningful.”

#### 3.2.2. Sense of belongingness

Most participants from all four cities reported a sense of belongingness between caregiver and care receiver as a significant contributing factor to positive caregiving. Here many participants emphasized the strengthening of family bonding and the development of a friendly relationship between caregiver and care receivers. Like a participant from Pune (Daughter, 36 years) said, “I always share all my information with my mother, and she shares everything with me. We are emotionally connected to each other,” Another participant from Prayagraj (Daughter-in-law, 45 years) said, “Through this caregiving role I have come closer to my mother-in-law. This has strengthened our relationship. She admires me for my hard work.” Under this theme, some caregivers also emphasized on building friendly relationships with their care receivers. Like a caregiver from Visakhapatnam (Daughter-in-law, 39 years) said, “While taking care of my parents-in-law, we have become good friends.” Similarly, a caregiver from Guwahati (Son, 48 years) said, “Me and my parents have become very good friends, we share every aspect of our lives with each other.”

#### 3.2.3. Personal growth

In the context of this theme, all caregivers from four cities shared that their caregiving role has made them more self-regulated, disciplined, flourished, and organized which in turn has contributed to their positive caregiving experiences. Like a participant from Pune (Daughter-in-law, 40 years) said, “Due to caregiving role, I learned how to manage my time in between my work and family.” Similarly, another caregiver from Guwahati (Daughter, 50 years) said, “This caregiving role is tough, but it also teaches lots of self-discipline, which I am learning while caring for my father.” Regarding personal growth, some participants have also reported that their caregiving role has made them more patient and self-regulated. Like a participant from Prayagraj (Daughter-in-law, 37 years) mentioned, “I do have a lot of stress issues, but when I think for a while and interact with my mother-in-law, I feel relaxed.” Similarly, another caregiver from Visakhapatnam (Son, 48 years) said, “My caregiving role for my parents has taught me to be patient and manage things well.”

#### 3.2.4. Empathetic understanding

Regarding the theme “Empathetic understanding,” most participants from Prayagraj and Visakhapatnam reported that due to their caregiving role, they have become more empathetic and can recognize care receiver’s emotions well and thus feel optimistic about it. Due to this, they can provide need-based emotional support to care receivers. Like participants from Prayagraj (Daughter, 38 years) said, “While taking care of my parents, I have become more understanding towards their needs. I am glad I am able to fulfill my responsibility as a daughter.” Similarly, another participant from Visakhapatnam (Daughter-in-law, 40 years) said, “People often talk about tussles between daughter-in-law and mother-in-law; this happens because of lack of understanding. In our case, I make special efforts to understand her situation and provide emotional support to my mother-in-law. She also appreciates this.”

Compared to the above, most participants from Pune and Guwahati emphasized that they can empathetically understand the challenges faced by their care receivers, work on resolving those challenges, and thus experience positive caregiving experiences. Like a caregiver from Pune (Daughter-in-law, 40 years) said, “My mother-in-law is very old, and she cannot walk on her own, so I have made it a habit that in the morning every day, I assist her in her walks. In this way, she meets other elders and interacts with them. She feels good about it, which gives me satisfaction.” Similarly, another participant from Guwahati (Daughter, 50 years) said, “As my parents are mostly staying at home, I help them connect with their friends and extended family via WhatsApp and Zoom. I know they like doing that. It makes me feel happy.”

## 4. Discussion

The findings of this study clearly indicate that informal caregiving, which is often considered burdensome, can be a flourishing experience for a family or informal caregiver, which not only contributes to their personal and spiritual growth but also strengthens their family bonding.

Based on the study’s findings, it can be said that the themes have been found to be associated with the PERMA model. All four themes for the Nature of Caregiving, namely Caregiver’s attitude, Care and compassion, Roles and responsibilities, and Beliefs and values, are related to the domains of the PERMA model like Positive Emotion (P), Engagement (E), Relationships (R), Meaning (M), and Accomplishment (A). In the case of the first theme, Caregiver’s attitude, many caregivers across four cities reported that their caregiving experiences had helped them develop positive attitudes, making life more meaningful and satisfying. This is strongly related to the “meaning” domain of the PERMA model and justifies that getting involved in a cause that matters can bring more meaning to life (38). Similar results were observed in the study by Sánchez-Izquierdo and associates (8).

In the case of the second theme, Care and compassion, most caregivers shared the positive feelings they experienced while providing care to their older adults parents or in-laws. The positive emotions experienced by most of these caregivers are hope, interest, joy, love, compassion, pride, amusement, and gratitude, which are very similar to the ones mentioned in the “positive emotion” domain of the PERMA model (33).

Similarly, through the third theme, roles and responsibilities, caregivers reported that their caregiving roles have added purpose to their life through engagement and involvement in the care receiver’s life. This is related to the “engagement” domain of the PERMA model, which emphasizes on being mindful of the present moment and focusing on the current endeavors (30). In other words, it means getting engrossed and absorbed in the task. Most participants across four cities reported getting completely absorbed in their caregiving role and doing that duty with full dedication which in turn contributes to their personal growth and family bonding.

Regarding the fourth theme, beliefs and values, most participants shared their beliefs and values about their caregiving roles and emphasized how it contributes to their well-being. Based on their lived experiences, most of them considered caregiving a pious and holy duty that would bring them good karma and help them attain their spiritual growth. This is very similar to the “meaning” domain, which is associated with having a purpose in life and guided by personal values. Some association can also be established with the domain of “accomplishment,” which means achievement, mastery, or competence, as many caregivers reported their caregiving role as a valued accomplishment in their life.

Thus, based on the findings and the above discussion, it can be concluded that caregiving can be a flourishing and positive experience among informal caregivers The attributes of positive caregiving will mainly comprise of caregiver’s positive attitude towards their life and caregiving role, care and compassion towards their care receivers, commitment towards their caregiving roles and responsibilities, and their profound beliefs and values which they associate with their caregiving duty.

After discussing the nature of positive caregiving, the next important step is to explore the determinants or contributing factors of positive caregiving among informal caregivers. The four significant themes that emerged as factors contributing to positive caregiving are “Meaningfulness in life,” “Sense of belongingness,” “Personal growth,” and “Empathetic understanding.” All these themes have a strong association with the PERMA model.

Regarding meaningfulness in life, most caregivers reported that they found their caregiving role significant and worthwhile. Thus, it has added more meaningfulness to their life. This is very similar to the “meaning” domain of the PERMA model, which is associated with doing things that give individuals a sense of purpose and worth. This sense of meaning can be different for everyone and is guided by personal values (48).

The second contributing factor to positive caregiving is sense of belongingness. In this regard, most caregivers mentioned that caregiving leads to the strengthening of family bonding and the building of friendly relationships. This highlights the “relationship” domain of the PERMA model, which relates to how people connect with others (48). Researchers have demonstrated that sharing good news or celebrating success builds solid bonds and better relationships (36). Caregivers have reported similar things in this study that while performing their caregiving role, they share their feelings, success, emotions, and anxieties with care receivers, which makes them feel good and relieved and builds their bonds.

The theme of personal growth emphasizes the growth and gains that caregivers have reported due to the caregiving role. Many shared that due to their caregiving role, they are leading a self-regulated and disciplined life and are experiencing various signs of personal growth like spiritual development, a sense of personal control, control of anger, and high self-esteem. These experiences are related to the “accomplishment” domain of the PERMA model, which emphasizes competence and personal growth (48).

Another important contributing factor is empathetic understanding. Most caregivers reported that as they spend maximum of their time with their care receivers, they have become empathetic towards them. This empathy is not only helping the care receivers but also serving the caregivers in experiencing positive emotions, building positive relationships, and finding meaningfulness in their roles. All these are essential determinants of flourishing (48).

Thus, based on the findings and the above discussion, we can say that positive caregiving is an important indicator of caregivers’ and care receivers’ well-being. The results suggest that when the caregivers looked at caregiving with a positive attitude by considering their caregiving role as pious and assigning meaningfulness to their act of caregiving, it not only made them connect with the care receivers on an emotional level but also contributed to their family bonding and personal growth. Findings also highlight that, in the context of older adults care receivers, positive caregiving in the family not only promotes healthy aging and reduces the percentage of older adults seeking institutional support but also contributes to building intergenerational bonding and making societies elder-friendly. The findings have implications for planning some action research, training, and counseling programs for promoting positive aspects of caregiving among informal caregivers.

## 5. Limitations

The study has certain limitations that need to be considered by researchers who wish to explore this area further. The study utilized a purposive sampling technique, which may not represent the entire population of caregivers of the older adults in India. The study was limited to only four cities in India, so the results may not be generalized to other regions. This study relied on self-reported data from caregivers, which may have been subject to social desirability or recall bias.

## 6. Areas for future research

Future studies could use a larger sample size and employ a mixed methods approach to triangulate qualitative findings with quantitative data results and provide a more comprehensive understanding of the positive aspects of caregiving. Future studies could also investigate the effectiveness of intervention programs that are designed to promote positive caregiving. This could include exploring the impact of training, support groups, or counseling programs on the well-being of caregivers and care receivers. It is also necessary to plan longitudinal studies to investigate how the dimensions of positive caregiving might change over time and to explore the factors that contribute to sustained positive caregiving practices among caregivers.

## Data availability statement

The raw data supporting the conclusions of this article will be made available by the authors, without undue reservation.

## Ethics statement

Ethical review and approval was not required for the study on human participants in accordance with the local legislation and institutional requirements. The patients/participants provided their written informed consent to participate in this study.

## Author contributions

NM conceptualized and designed the study, reviewed, and analyzed the data. RD reviewed the data and checked the methodological quality. AT reviewed the data and edited the manuscript. MS reviewed the data and guided the field investigators along with NM to conduct interviews. All the authors contributed to data collection, interpretation of the data, manuscript writing and review of the manuscript.

## Conflict of interest

The authors declare that the research was conducted in the absence of any commercial or financial relationships that could be construed as a potential conflict of interest.

## Publisher’s note

All claims expressed in this article are solely those of the authors and do not necessarily represent those of their affiliated organizations, or those of the publisher, the editors and the reviewers. Any product that may be evaluated in this article, or claim that may be made by its manufacturer, is not guaranteed or endorsed by the publisher.

## References

[1] United Nations. Department of Economic and Social Affairs, Population Division of the United Nations Department of Economic and Social Affairs (UN DESA). (2022)

[2] NSO. Elderly in India, National Statistical Office In: Ministry of Statistics & Programme Implementation, Government of India. New Delhi: (2021)

[3] Gustafsson L-K Morell IA Johansson C De S. Informal caregiving from the perspectives of older people living alone in India. Int J Older People Nursing. (2022) 17:e12468. doi: 10.1111/OPN.12468, PMID: 35466547 PMC9787525

[4] Plöthner M Schmidt K de Jong L Zeidler J Damm K. Needs and preferences of informal caregivers regarding outpatient care for the elderly: a systematic literature review. BMC Geriatr. (2019) 19:82. doi: 10.1186/S12877-019-1068-4, PMID: 30866827 PMC6417014

[5] Ugargol AP Hutter I James KS Bailey A. Care needs and caregivers: associations and effects of living arrangements on caregiving to older adults in India. Ageing Int. (2016) 41:193–213. doi: 10.1007/S12126-016-9243-9, PMID: 27340308 PMC4877410

[6] Mitchell GJ. The lived experience of taking life day-by-day in later life: Research guided by Parse’s emergent method. Nurs Sci Q. (1990) 3:29–36. doi: 10.1177/089431849000300108

[7] Dhar RL. Caregiving for elderly parents: a study from the Indian perspective. Home Health Care Manag Pract. (2012) 24:242–54. doi: 10.1177/1084822312439466

[8] Sánchez-Izquierdo M Prieto-Ursúa M Caperos JM. Positive aspects of family caregiving of dependent elderly. Educ Gerontol. (2015) 41:745–56. doi: 10.1080/03601277.2015.1033227

[9] Roth DL Fredman L Haley WE. Informal caregiving and its impact on health: A reappraisal from population-based studies. The Gerontologist. (2015) 55:309–19. doi: 10.1093/geront/gnu177, PMID: 26035608 PMC6584119

[10] Beach SR Schulz R Williamson GM Miller LS Weiner MF Lance CE. Risk factors for potentially harmful informal caregiver behavior. J Am Geriatr Soc. (2005) 53:255–61. doi: 10.1111/j.1532-5415.2005.53111.x, PMID: 15673349

[11] Ampalam P Gunturu S Padma V. A comparative study of caregiver burden in psychiatric illness and chronic medical illness. Indian J Psychiatry. (2012) 54:239–43. doi: 10.4103/0019-5545.102423, PMID: 23226847 PMC3512360

[12] Delalibera M Presa J Barbosa A Leal I. Burden of caregiving and its repercussions on caregivers of end-of-life patients: a systematic review of the literature. Cien Saude Colet. (2015) 20:2731–47. doi: 10.1590/1413-81232015209.09562014, PMID: 26331505

[13] Dunkin JJ Anderson-Hanley C. Dementia caregiver burden: a review of the literature and guidelines for assessment and intervention. Neurology. (1998) 51:S53–60. doi: 10.1212/WNL.51.1_SUPPL_1.S53 9674763

[14] Thakur V Nagarajan P Rajkumar RP. Coping and burden among caregivers of patients with major mental illness. Indian J Soc Psychiatr. (2022) 0:01. doi: 10.4103/IJSP.IJSP_160_20

[15] Li Q Loke AY. The positive aspects of caregiving for cancer patients: a critical review of the literature and directions for future research. Psycho-Oncology. (2013) 22:2399–407. doi: 10.1002/PON.3311, PMID: 23712938

[16] López J López-Arrieta J Crespo M. Factors associated with the positive impact of caring for elderly and dependent relatives. Arch Gerontol Geriatr. (2005) 41:81–94. doi: 10.1016/J.ARCHGER.2004.12.001, PMID: 15911041

[17] Sarabia-Cobo C Sarriá E. Satisfaction with caregiving among informal caregivers of elderly people with dementia based on the salutogenic model of health. Appl Nurs Res. (2021) 62:151507. doi: 10.1016/J.APNR.2021.151507, PMID: 34815003

[18] Cohen CA Colantonio A Vernich L. Positive aspects of caregiving: rounding out the caregiver experience. Int J Geriatr Psychiatry. (2002) 17:184–8. doi: 10.1002/gps.561, PMID: 11813283

[19] Lwi SJ Ford BQ Levenson RW. Cultural differences in caring for people with dementia: a pilot study of concern about losing face and loneliness in Chinese American and European American caregivers. Clin Gerontol. (2023) 46:207–22. doi: 10.1080/07317115.2022.2137448, PMID: 36309843 PMC9928887

[20] Birtha M Holm K. WHO CARES? Study on the challenges and needs of family carers in Europe. Brussels: COFACE (2017).

[21] Prasad BD Indira Rani N. Older persons, and caregiver burden and satisfaction in rural family context. Indian J Gerontol. (2007) 21:216–32.

[22] Cejalvo E Martí-Vilar M Merino-Soto C Aguirre-Morales MT. Caregiving Role and Psychosocial and Individual Factors: A Systematic Review. Healthcare. (2021) 9:1690. doi: 10.3390/healthcare9121690, PMID: 34946416 PMC8700856

[23] Sharma N Chakrabarti S Grover S. Gender differences in caregiving among family - caregivers of people with mental illnesses. World Journal of Psychiatry. (2016) 6:7–17. doi: 10.5498/wjp.v6.i1.7, PMID: 27014594 PMC4804270

[24] Ajay S Kasthuri A Kiran P Malhotra R. Association of impairments of older persons with caregiver burden among family caregivers: Findings from rural South India. Arch Gerontol Geriatr. (2017) 68:143–8. doi: 10.1016/J.ARCHGER.2016.10.003, PMID: 27810662

[25] Puzhakkal JC Kallivayalil RA Sudhakar S. Assessment of caregiver burden and their quality of life at a tertiary care center: A cross-sectional study. Indian J Soc Psychiatr. (2019) 35:88. doi: 10.4103/IJSP.IJSP_119_17

[26] Felipe SG Oliveira CE d S Silva CRDT Mendes PN Carvalho KM d Lopes Silva-Júnior F. Anxiety and depression in informal caregivers of dependent elderly people: an analytical study. Rev Bras Enferm. (2020) 73:e20190851. doi: 10.1590/0034-7167-2019-0851, PMID: 32965315

[27] Jones SBW Whitford HS Bond MJ. Burden on informal caregivers of elderly cancer survivors: risk versus resilience. J Psychosoc Oncol. (2015) 33:178–98. doi: 10.1080/07347332.2014.1002657, PMID: 25658457

[28] Srivastava G Tripathi RK Tiwari SC Singh B Tripathi SM. Caregiver burden and quality of life of key caregivers of patients with dementia. Indian J Psychol Med. (2016) 38:133–6. doi: 10.4103/0253-7176.178779, PMID: 27114625 PMC4820552

[29] Alshammari SA Alzahrani AA Alabduljabbar KA Aldaghri AA Alhusainy YA Khan MA. The burden perceived by informal caregivers of the elderly in Saudi Arabia. J Fam Community Med. (2017) 24:145–50. doi: 10.4103/JFCM.JFCM_117_16, PMID: 28932158 PMC5596626

[30] Seligman MEP. Flourish: a visionary new understanding of happiness and well-being Free Press (2012).

[31] Khaw D Kern M. A cross-cultural comparison of the PERMA model of well-being. Undergraduate J Psychol. (2014) 8:10–23.

[32] Kern ML Waters LE Adler A White MA. A multidimensional approach to measuring well-being in students: Application of the PERMA framework. J Posit Psychol. (2015) 10:262–71. doi: 10.1080/17439760.2014.936962, PMID: 25745508 PMC4337659

[33] Fredrickson BL. The role of positive emotions in positive psychology: The broaden-and-build theory of positive emotions. Am Psychol. (2001) 56:218–26. doi: 10.1037/0003-066X.56.3.218 11315248 PMC3122271

[34] Tugade MM Fredrickson BL. Resilient individuals use positive emotions to bounce back from negative emotional experiences. J Pers Soc Psychol. (2004) 86:320–33. doi: 10.4103/IJSP.IJSP_160_20, PMID: 14769087 PMC3132556

[35] Seligman MEP Steen TA Park N Peterson C. Positive psychology progress: empirical validation of interventions. Am Psychol. (2005) 60:410–21. doi: 10.1037/0003-066X.60.5.410 16045394

[36] Siedlecki KL Salthouse TA Oishi S Jeswani S. The relationship between social support and subjective well-being across age. Soc Indic Res. (2014) 117:561–76. doi: 10.1007/s11205-013-0361-4, PMID: 25045200 PMC4102493

[37] Kashdan TB Mishra A Breen WE Froh JJ. Gender differences in gratitude: examining appraisals, narratives, the willingness to express emotions, and changes in psychological needs. J Pers. (2009) 77:691–730. doi: 10.1111/j.1467-6494.2009.00562.x, PMID: 20078735

[38] Tang J Li X-c Zhang X. The Eudemonic Wellbeing of Volunteers in a Public Health Emergency: COVID-19 in China. Front Psychol. (2022) 13:903147. doi: 10.3389/fpsyg.2022.903147 35719588 PMC9200989

[39] Valle RS King M Halling S. An introduction to existential-phenomenological thought in psychology In: Existential-phenomenological perspectives in psychology. Boston, MA: Springer (1989). 3–16.

[40] Creswell JW Miller DL. Determining validity in qualitative inquiry. Theory Pract. (2000) 39:124–30. doi: 10.1207/s15430421tip3903_2

[41] Nakkeeran N. Qualitative Research Methodology: epistemological foundation and research procedures. Indian J Soc Work. (2017) 67:2006–104.

[42] Miles MB Michael Huberman A. Qualitative data analysis: An expanded sourcebook SAGE Publications (1994).

[43] Brink HI. Validity and reliability in qualitative research. Curationis." (1993) 31:35–8. 10.4102/curationis.v16i2.1396 8375009

[44] Lincoln Yvonna S. Guba Egon G. Naturalistic inquiry. SAGE Publications, (1985), 9, 438–439

[45] Braun V Clarke V. Using thematic analysis in psychology. Qual Res Psychol. (2006) 3:77–101. doi: 10.1191/1478088706qp063oa

[46] Boyatzis RE. Transforming qualitative information: thematic analysis and code development SAGE Publications (1998).

[47] Tuckett AG. Applying thematic analysis theory to practice: A researcher’s experience. Contemp Nurse. (2005) 19:75–87. doi: 10.5172/conu.19.1-2.75, PMID: 16167437

[48] LIFE Research Institute Investigating Flourishing in Caregivers of Older Adults. A compendium of protocols designed for research. Ottawa: Canada. University of Ottawa (2021).

